# Exogenous biostimulant bee-honey solution improves *Triticum aestivum* L. tolerance to salt stress by modulating physio-biochemical responses and upregulation of salinity-related genes

**DOI:** 10.3389/fpls.2025.1761667

**Published:** 2026-01-19

**Authors:** Asma Algasem, Sameera Alghamdi, Hameed Alsamadany

**Affiliations:** 1 Department of Biological Sciences, Faculty of Science, King Abdulaziz University, Jeddah, Saudi Arabia; 2 Department of Biology, Faculty of Science, Al-Baha University, Al-Baha, Saudi Arabia

**Keywords:** antioxidant response, biostimulants, cereals, gene expression, oxidative stress

## Abstract

**Introduction:**

Salinity is a major global constraint to wheat productivity, primarily due to oxidative damage, osmotic imbalance, and ionic toxicity. This study evaluated the potential of bee-honey solution (BHS) as a natural biostimulant to mitigate salinity stress effects in wheat (cv. Yecora Rojo).

**Methods:**

Plants were treated with 0, 100, 150, and 200 mM NaCl, with foliar application of BHS (0, 1.0, and 1.5%). BHS potential influences on plant growth, physio-biochemical indices, antioxidant defense system, and gene expression under four salinity levels were evaluated.

**Results:**

Salt stress at 200 mM, decreased plant height by 25%, leaf area by 14%, and biomass by 28% compared to control, while chlorophyll content reduced by 63%, and the Na^+^/K^+^ ratio rose by 206%. Besides, ROS levels inclined markedly, with H_2_O_2_ (+ 125%), O_2_•^−^ (+ 100%), and MDA (+ 129%) accumulation relative to non-stressed plants. Foliar application of BHS, particularly at 1.5%, significantly mitigated these effects: plant height increased by 37%, leaf area by 43%, and biomass by 52% compared to stressed-control plants. Chlorophyll content increased by 73%, RWC rose by 25%, and the Na^+^/K^+^ ratio decreased by 23%. Besides, BHS significantly reduced H_2_O_2_ (- 17%), O_2_•^−^ (- 18%), and MDA (- 31%) levels while increasing antioxidant enzyme activities: SOD (+ 33%), POD (+ 86%), and CAT (+ 38%). Proline content increased by 25% through *TaP5CS* upregulation, while GB decreased by 29%, indicating regulated osmolytic balance. Gene expression analysis showed strong upregulation of *TaPOD-D1* (+ 4.1-fold), *TaSOD2* (+ 3.8-fold), *TaCAT1* (+ 3.6-fold), *TaNHX2* (+ 2.9-fold), and *TaHKT1;4* (+ 3.2-fold) in BHS-treated plants under salt stress. Multivariate analyses; PCA and heatmap confirmed close association of BHS treatments with increased physiological, biochemical, and molecular responses, particularly under high salinity level.

**Discussion & conclusion:**

The present findings prove that BHS is an effective, eco-friendly biostimulant that imparts salt resilience to wheat by integrating morphological, physiological, biochemical, and molecular mechanisms.

## 1 Introduction

Common or bread wheat (*Triticum aestivum* L.), an annual grass, is among the most devoured cereal grains worldwide, beside maize and rice. It belongs to the Poaceae family and represents approximately 95% of the worldwide grown wheat (Moshawih et al., 2022; Khalid et al., 2023). Commonly, wheat is used in baking and cooking and valued for its content of carbohydrates and other vital components such as dietary fiber, lipids, proteins, minerals, terpenoids, phytochemicals and vitamins (Zhong et al., 2023). Amongst wheat widespread cultivars, *T. aestivum* cv. Yecora Rojo has distinctive agronomic characteristics. It is a red spring hard wheat registered in 1975 in California, United States (Qualset et al., 1985).

Salt stress, an abiotic stress, is a major environmental issue and one of the major causes of agricultural yield loss. Wheat, among other field crops, is more sensitive to salinity stress which is known to be the most negatively affecting factor on wheat quality and productivity. According to the severity of salinity, it can cause low productivity or complete crop loss (Seleiman et al., 2022). High Na^+^ and Cl^-^ concentrations in the soil alter the normal mechanism of germination, affecting adversely the growth, development, and economic yield of the plant. Previous investigations about salinity impacts on bread wheat have revealed that soil salinity affects detrimentally shoot and root length, plant height, number of leaves per plant, fresh and dry weight, leaf area, root/shoot ratio, length and number of spikes, and grains number spike^-1^ (El Sabagh et al., 2021). Additionally, salt stress affects significantly the physiological and biochemical parameters of the plant such as water potential, tissue water content, water use efficiency, root hydraulic conductance, cell turgidity, starch content, total kernel weight, and nutritional quality attributes (Khataar et al., 2018; Dadshani et al., 2019; Nadeem et al., 2020).

Bee-honey mainly has sugars, amino acids, organic acids, minerals, and phenolic compounds that serve as antioxidants (Da Silva et al., 2016; Cianciosi et al., 2018). Besides, GC-MS and HPLC studies confirmed these bioactive compounds (BACs), confirming bee-honey as a natural plant growth promoter (Manyi-Loh et al., 2011; Chua et al., 2012). Bee-honey solution (BHS) has been reported as a natural foliar biostimulant that improves plant growth and stress tolerance by increasing photosynthesis, antioxidant activity, osmo-protectants concentration, and nutrient balance. Its beneficial impacts have been reported in drought-stressed common bean (*Phaseolus vulgaris*) (Alghamdi et al., 2022), nitrogen-stressed common bean (Belal et al., 2023), faba bean (*Vicia faba*) under drought (Rady et al., 2021), and sage (*Salvia officinalis*) (Moussa et al., 2024). These results indicate that BHS may serve as an eco-friendly tool to mitigate abiotic stress and increase crop productivity.

Wheat sustains world food security but is highly vulnerable to drought and salinity stresses that reduce photosynthesis, impair nutrient-uptake, and reduce grain yield (Gupta et al., 2008; Farooq et al., 2014). Current stress-management strategies largely depend on synthetic inputs, which are costly and environmentally unsustainable (Shiferaw et al., 2013). Hence, there is a strong need for natural and eco-friendly alternatives that can increase wheat tolerance to abiotic stresses.

Components such as soluble sugars, organic acids, proline, essential nutrients, ascorbic acid (AsA), and B-group vitamins have successfully been applied individually to support plant performance under stressed conditions (Mekdad and Rady, 2016; Zahid et al., 2018; Ould Said et al., 2021; Zamanipour, 2021; Al-Taweel et al., 2022; Selem et al., 2022; Hemida et al., 2023). Via utilizing BHS, which is a complex combination of organic forms of components, all these vital components are combinedly applied giving a multi-mechanism effect including osmo-regulation, organic acids, nutrition, and antioxidant systems. Consequently, a multi-level effect is generated via different signaling pathways which affect plant responses to adapt to environmental stresses.

BHS has recently attracted attention as a foliar biostimulant because of its rich composition of sugars, amino acids, minerals, vitamins, and antioxidants. Furthermore, noteworthy outcomes have been reported in legumes, where BHS enhanced stress tolerance by modulating osmoprotectants, antioxidants, and nutrient balance (Rady et al., 2021; Alghamdi et al., 2022), and in aromatic and horticultural crops such as *Salvia officinalis*, basil, and chili pepper, where BHS improved stress tolerance and secondary metabolism (Abou-Sreea et al., 2021; Moussa et al., 2024; Sadou et al., 2025). Despite this, no comprehensive study has elucidated the potential of BHS in wheat, one of the world’s most stress-sensitive cereal crops whose yield stability is threatened by frequent drought and salinity episodes. Although, previous works on the effects of foliar nourishment on *T. aestivum* plants grown under salt stress conditions have been documented (Ayman et al., 2024; Tajdari et al., 2024), so far, no available research has addressed *T. aestivum* responses triggered by the foliar nourishment with BHS under normal and/or stress conditions. The present study, therefore, aims to highlight the potential usefulness of BHS foliar application in *T. aestivum* cv. Yecora Rojo plants to ameliorate salinity stress impacts, contributing to the recent growing body of evidence that indicates the BHS efficacy towards abiotic stresses.

## 2 Material and methods

### 2.1 Grains collection and treatments application

Grains of cv. Yecora Rojo were obtained from the National Seed and Agriculture Services Company (BUTHOR), Riyadh, Saudi Arabia. Prior to sowing, grains were surface sterilized utilizing sodium hypochlorite (1%) for 2 min. Grains were then washed with distilled water and left to dry overnight at room temperature.

The experiment was conducted in a shade house at King Abdulaziz University, Jeddah, Saudi Arabia. The experiment was performed under normal conditions of temperatures (25.24 ± 0.25 °C), relative humidity (49.55 ± 1.02%), daylight length (11.13 ± 0.00 h), and total rainfall (0.60 ± 0.57 mm) (data (means ± SE) were obtained from the National Centre for Meteorology, Jeddah, KAIA station). Regarding light intensity, natural sunlight was suitable for wheat plants growth during the period of the experiment.

Previous works have explored the effect of different salinity levels on wheat plants. Sodium chloride concentrations such as 100, 120, 150, and 200 mM were used to induce salinity in wheat plants (Seleiman et al., 2022). In the current work, salt stress was induced at 100, 150, and 200 mM NaCl.

Two experimental factors were included in the experiment, which were salinity stress levels (0, 100, 150, and 200 mM NaCl) and BHS foliar treatments (0, 1.0, and 1.5%) with factorial arrangement of 4 × 3 × 3 representing stress conditions, BHS treatments, and replications, respectively. The experiment was initiated utilizing 36 plastic pots (30 cm in diameter each) loaded with 5 kg of the soil each, with 10 planted grains per pot. Tap water was utilized to irrigate all pots. Once the seedlings reached the three-leaves stage, two actions were taken. The first was reducing the number of seedlings to five per pot. The second was dividing the pots into four groups for the application of salinity stress treatments. For each of these groups, foliar spray with BHS concentrations was applied once every week. Until the completion of the experiment, a total of 3 × foliar sprays were done. Different BHS treatments were sprayed at 50, 64, and 73 mL pot^-1^ for the three sprays, respectively. Pots were kept in a randomized complete block design (RCBD) and the experiment duration was 60 days.

### 2.2 Bee-honey solution preparation

The alfalfa (*Medicago sativa* L.) honey sample was obtained from the Beekeepers Cooperative Association, Al-Qassim, Saudi Arabia. The physiochemical properties of the used fresh raw alfalfa honey are shown in **Table 1**. A preliminary experiment with five BHS concentrations (0 (control), 0.5, 1.0, 1.5, and 2.0%) were used for selecting the most effective BHS concentrations. For the preparation of 1.0 and 1.5% solutions of BHS, 10 and 15 g of the honey sample were well dissolved in 1 L of distilled water each, respectively. Tween-20 (polysorbate 20; 0.1%) was used as a surfactant to achieve the optimal penetration of BHS to the leaf tissue. Prepared BHS solutions were used as foliar nourishment after 20 min from preparation. Sprays with BHS were performed to run-off early morning utilizing a hand atomizer.

**Table 1:** Physiochemical properties of *M. sativa* honey sample.

Property/Component	Unit	Value
Moisture	%	16.6
pH		4.2
Electrical conductivity	mS cm^-1^	0.58
Ash	%	0.03
Organic acids		0.093
Mineral nutrients:		
Potassium (K)	mg kg^-1^ FW	624.51
Calcium (Ca)	mg kg^-1^ FW	289.88
Iron (Fe)	mg kg^-1^ FW	224.71
Magnesium (Mg)	mg kg^-1^ FW	107.97
Phosphorus (P)	mg kg^-1^ FW	44.74
Manganese (Mn)	mg kg^-1^ FW	13.61
Zinc (Zn)	mg kg^-1^ FW	5.84
Osmoprotectants:		
Fructose	%	43.0
Glucose	%	33.0
Sucrose	%	2.0
Protein	%	0.37
Proline	mg kg^-1^ FW	8.7
Antioxidants and vitamins:		
C (Ascorbic acid)	mg kg^-1^ FW	4.9
B1 (Thiamine)	mg kg^-1^ FW	5.1
B2 (Riboflavin)	mg kg^-1^ FW	1.2
B3 (Niacin)	mg kg^-1^ FW	6.2
B5 (Pantothenic acid)	mg kg^-1^ FW	8.9
B6 (Pyridoxine)	mg kg^-1^ FW	11.2
B9 (Folic acid)	µg kg^-1^ FW	75
DPPH antioxidant activity (IC_50_)	Parts per million	0.1

### 2.3 Collection of data

#### 2.3.1 Growth qualities

Growth measurements including plant height, leaves number plant^-1^, leaf area, and shoot fresh and dry weights were measured after two weeks from the last treatment with BHS treatments.

#### 2.3.2 Physiological qualities

Leaves content of chlorophyll was estimated as per the procedure mentioned in Mahmood et al. (2016). Fresh leaf samples (0.5 g) were soaked in acetone (80%) in a shaker until the complete bleaching of the leaves. Centrifugation was then taken place for the resulting extract at 13,000 rpm for 10 min. Then, the liquid acetone extract was used to record the total chlorophyll (a and b) levels at 663 and 645 nm absorbance utilizing a spectrophotometer.

Cell membrane stability (CMS) was evaluated based on the differences of EC measurements of the leaf tissue solution prior and after heating (Alghabari et al., 2021). Leaf blades were divided into pieces (100 mg each) and placed in two tubes, each having 20 mL deionized water (one sample per tube). Then, one tube was incubated at 40 °C for 30 min to record EC1. The other tube was boiled at 100 °C for 10 min to record EC2. On completion of EC1 and EC2 recording, the following equation was used to calculate CMS:


CMS (%) =1− [EC1÷EC2] ×100

Determination of relative water content (RWC) was based on determining the fresh weight (FW), turgid weight (TW), and dry weight (DW) of leaf discs as described by Osman and Rady (2014). The following equation was used to calculate RWC:


RWC (%)= [(FW−DW)÷(TW −DW)]×100

For water potential (Ψ_w_) and osmotic potential (Ψ_s_) measurement, the method detailed by Sattar et al. (2020) was adopted. Regarding Na^+^/K^+^ content, it was estimated from leaf samples using the method outlined by Hniličková et al. (2019).

#### 2.3.3 Biochemical qualities

For assessing the activities of superoxide dismutase (SOD), peroxidase (POD), and catalase (CAT), commercially available kits were used. Utilizing the SOD1 ELISA kit (product No. MBS283325, MyBioSource, United States), the activity of SOD was determined accordingly with the manufacturer’s guidelines. As for POD activity evaluation, the peroxidase activity analytical kit (product No. E-BC-K227-S, Elabscience, United States) was used based on the provided protocol. CAT activity was evaluated using the catalase analytical kit (product No. MBS8243260, MyBioSource, United States) in accordance with the provided protocol. Representative standard absorbance curves were then used to quantify the activities of antioxidant enzymes, and the results were expressed as enzyme units.

Glycine betaine (GB) content was determined spectrophotometrically under acidic conditions following the method of Valadez-Bustos et al. (2016). The colorimetric method described in Forlani and Funck (2020) was utilized to determine the free proline content.

The peroxidation level of lipids was assessed as malondialdehyde (MDA) content. Using a spectrophotometer, the pink chromogen that was formed as a consequence of the reaction between MDA and thiobarbituric acid (TBA) was measured at 532 nm. A standard curve was then used to calculate MDA amount in the sample and the results were interpreted as nmol g^-1^ FW (Reilly and Aust, 1999). To estimate superoxide anion radicle (O_2_•^−^) content, the method outlined by Ajiboye et al. (2016) was used. Hydrogen peroxide (H_2_O_2_) content was estimated based on the methodology of Velikova et al. (2000).

#### 2.3.4 Molecular qualities

For the isolation of RNA from the plant samples, the Qiagen RNeasy kit (Qiagen, United States) was used according to the procedure outlined by Li et al. (2018). Thereafter, 2 µg of RNA was used to generate a cDNA library. As for qRT-PCR analysis, the SYBR Green 1 master kit was employed as per the manufacturer’s instructions. In addition, genes expression was standardized utilizing the Actin-expressing gene (Vradi03g00210). The primers used are shown in **Table 2**.

**Table 2:** List of primers used for qRT-PCR analysis.

Gene	Primer	Primer sequence
*TaPOD-D1*	Forward	AGCACACAAGGAGAGAGGAG
	Reverse	AAGAGGCACGCGGTAGTCG
*TaSOD2*	Forward	CGCAGGACAACCAATGGACC
	Reverse	CGGAGGCACACTAGGCATCC
*TaCAT1*	Forward	GGCCGCGCCGGAAACTGC
	Reverse	CGGGAACGAGAGGGCGAGAAAGA
*TaHKT1;4*	Forward	AGCAAGCTGAAGTTGAGGGG
	Reverse	AGAGTTGTGACAGAGCCGTG
*TaNHX2*	Forward	CTCAAGGGTGACTACCAAGCA
	Reverse	CCAATGCATCCATCCCGAC
*TaP5CS*	Forward	GAAGGCTCTTATGGGTGTACTCAA
	Reverse	TAAAAGACCTTCAACACCCACAGG
*TaBAS1*	Forward	TATTCATCATTGACAAGGAGGG
	Reverse	GGTCAGGCTTCATCGACTTTT
*TaFER-5B*	Forward	GCGTGGACCGTTGCTGCAACT
	Reverse	GGGCATCGCCTTTCTCAGCA
*TaACTIN*	Forward	TACTCCCTCACAACAACC
	Reverse	GCTCCTGCTCATAATCAAG

### 2.4 Statistical analysis

Data were analyzed based on the Statistix 8.1 software (McGraw-Hill, 2008) to determine the significant differences between treatments at a significant level of 5%. In addition, RStudio version 1.3.959 (RStudio Team, 2020) was utilized for the principal component analysis (PCA), correlation, and heatmap analyses.

## 3 Results

### 3.1 Growth qualities

**Table 3** provides an overview of the huge variation in growth characteristics of plants owing to the interaction influence of NaCl and BHS. The plant height and leaves number plant^-1^ traits were decreased significantly with rising concentrations of NaCl. As salinity level increased from S1 (control) to S2, S3, and S4, the plant height was decreased by 21.14, 20.61, and 25.10%, respectively, while leaves number plant^-1^ was reduced by 7.79% under S3 and S4 salinity levels. Other growth traits depicted negative changes due to salinity stress impact. However, foliar-applied T2 and T3 of BHS to plants subjected to S2 and S3 of salinity levels increased significantly the plant height, while at S1 and S4 salinity levels, T3 treatment increased significantly this trait. As to the leaves number plant^-1^, both BHS concentrations T2 and T3 increased the leaves number per plant significantly under S3 level of salinity, while treatment of T3 applied to plants grown under S2 and S4 salinity levels increased it significantly. In relation to the leaf area, both BHS concentrations (T2 and T3) increased the leaf area significantly under S1, S2, and S3 salinity levels, while T3 increased it significantly under S4 level. With respect to the shoot fresh weight, T3 concentration of BHS increased the shoot fresh weight significantly in S2, S3, and S4-stressed plants, while T2 concentration increased it significantly at S2 level of salinity. About shoot dry weight, T2 concentration of BHS increased this trait significantly at S1 level of salinity, whereas T3 concentration increased the same trait significantly at S3 and S4 levels of salinity.

**Table 3:** Interaction effect of foliar application with BHS treatments (T1:0, T2: 1.0, and T3: 1.5%) and various levels of salinity (S1: 0, S2: 100, S3: 150, and S4: 200 mM NaCl) on growth qualities of *T. aestivum* cv. Yecora Rojo plants.

Treatments	Plant height (cm)	No. of leaves plant^-1^	Leaf area (cm^2^)	Shoot FW (g)	Shoot DW (g)
S1 × T1	7.57 ± 0.33^bc^	5.78 ± 0.15^abc^	2.83 ± 0.13^efg^	0.29 ± 0.08^abc^	0.06 ± 0.00^bc^
S1 × T2	8.09 ± 0.24^ab^	6.00 ± 0.00^ab^	4.24 ± 0.27^a^	0.34 ± 0.02^a^	0.09 ± 0.01^a^
S1 × T3	8.53 ± 0.45^a^	6.11 ± 0.11^a^	3.78 ± 0.25^ab^	0.33 ± 0.01^a^	0.08 ± 0.00^ab^
S2 × T1	5.97 ± 0.19^d^	5.56 ± 0.18^cd^	2.49 ± 0.15^fg^	0.22 ± 0.01^c^	0.07 ± 0.01^abc^
S2 × T2	7.01 ± 0.40^c^	5.67 ± 0.17^bcd^	3.20 ± 0.07^cde^	0.31 ± 0.02^ab^	0.08 ± 0.01^ab^
S2 × T3	7.24 ± 0.42^bc^	6.00 ± 0.00^ab^	3.07 ± 0.18^de^	0.31 ± 0.02^ab^	0.08 ± 0.01^ab^
S3 × T1	6.01 ± 0.21^d^	5.33 ± 0.17^d^	2.37 ± 0.17^g^	0.22 ± 0.03^c^	0.05 ± 0.00^c^
S3 × T2	7.52 ± 0.22^bc^	6.00 ± 0.00^ab^	3.18 ± 0.17^cde^	0.27 ± 0.01^abc^	0.07 ± 0.00^abc^
S3 × T3	7.91 ± 0.20^ab^	6.00 ± 0.00^ab^	3.65 ± 0.35^bc^	0.32 ± 0.03^ab^	0.09 ± 0.01^a^
S4 × T1	5.67 ± 0.28^d^	5.33 ± 0.17^d^	2.44 ± 0.10^fg^	0.21 ± 0.03^c^	0.06 ± 0.01^bc^
S4 × T2	6.06 ± 0.27^d^	5.67 ± 0.17^bcd^	2.97 ± 0.14^def^	0.24 ± 0.01^bc^	0.07 ± 0.01^abc^
S4 × T3	7.78 ± 0.22^abc^	6.00 ± 0.00^ab^	3.48 ± 0.14^bcd^	0.32 ± 0.02^ab^	0.09 ± 0.01^a^

Values (means ± SE) followed by different superscripts are significantly different at *P* ≤ 0.05. S indicates salinity conditions while T indicates BHS treatments. FW means fresh weight and DW means dry weight.

### 3.2 Physiological qualities

**Figure 1:**
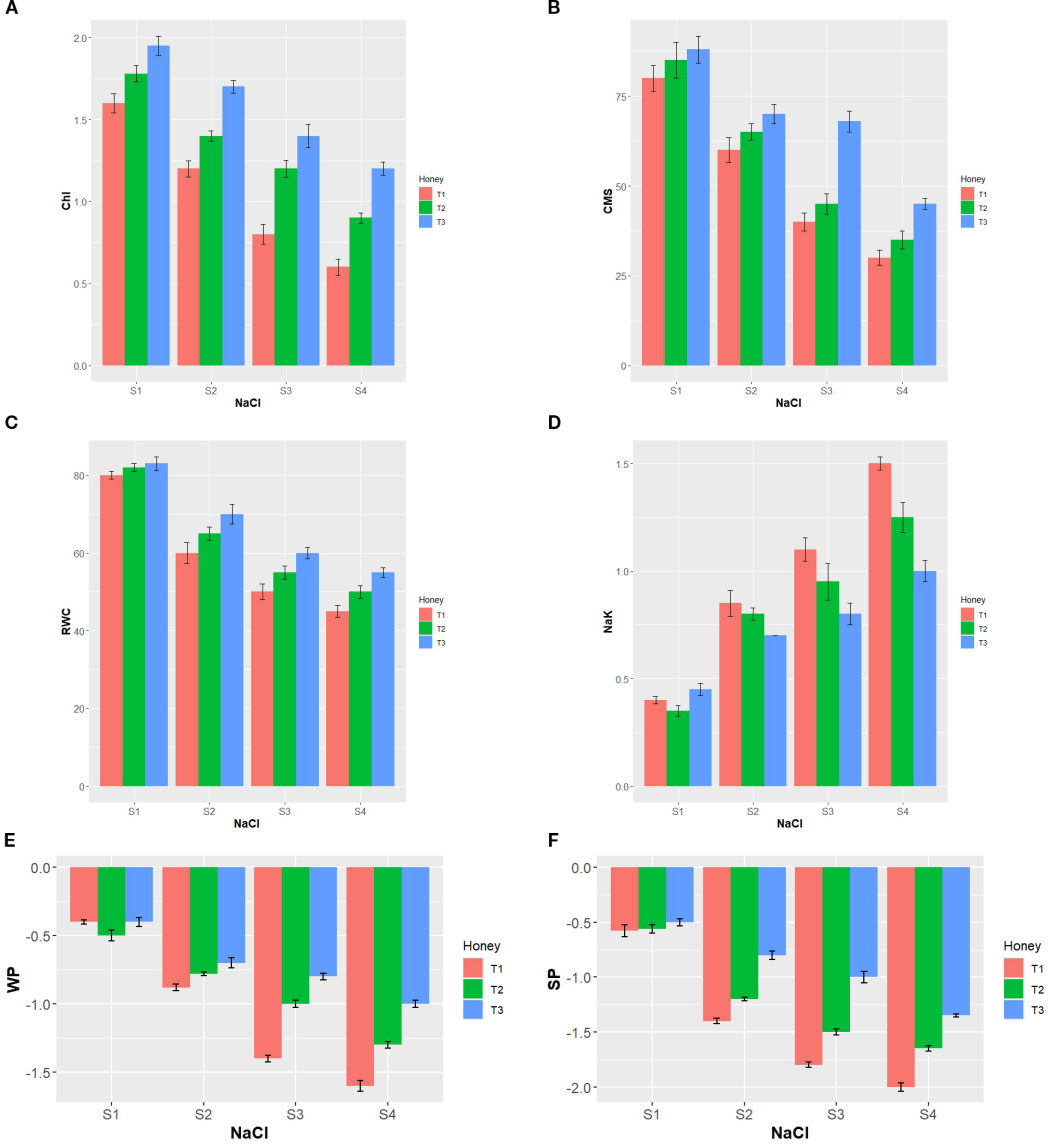
Interaction effect of foliar application with BHS treatments (T1: 0, T2: 1.0, and T3: 1.5%) and various levels of salinity (S1: 0, S2: 100, S3: 150, and S4: 200 mM NaCl) on physiological qualities of wheat (*Triticum aestivum* L., cv. Yecora Rojo). **(A)** Chl, chlorophyll content (g kg^-1^), **(B)** CMS, cell membrane stability (%), **(C)** RWC, relative water content (%), **(D)** Na^+^/K^+^ ratio, **(E)** WP, water potential (MPa), and **(F)** SP, osmotic potential (MPa).

Under the effect of salinity stress (S2, S3, and S4), chlorophyll and CMS were decreased significantly. The most adverse effect was noticed under S4 level of salinity which decreased these traits by 63.33 and 55.13%, respectively. However, foliar application with T2 and T3 manifested considerable increase in chlorophyll and CMS at all four levels of salinity. The best outcomes were yielded by T3 concentration which improved chlorophyll content by 26.67, 40.35, 71.43, and 72.73%, and CMS by 10.26, 23.64, 71.05, and 71.43% under S1, S2, S3, and S4, respectively, compared with the corresponding control (**Figures 1A, B**).

Variations in plant water affined parameters RWC, Ψ_w_, and Ψ_s_ due to the combined influence of salinity and BHS are set out in **Figures 1C, E, F**. Salt stress motivated by the addition of 100, 150, and 200 mM NaCl decreased significantly RWC, Ψ_w_, and Ψ_s_ of plants. The most obvious effects were noticed in plants grown under S4 (200 mM NaCl) which decreased RWC, Ψ_w_, and Ψ_s_ by 48.72, 265.85, and 221.43%, respectively. Conversely, under stressed conditions (from S2 to S4), the application of BHS treatments T2 and T3 increased substantially RWC, Ψ_w_, and Ψ_s_ of plants, compared to the corresponding control. Under unstressed circumstances (S1), the application of T2 and T3 increased RWC significantly (by 2.56 and 3.85%, respectively), while for Ψ_s_, T3 was more effective.

Analysis of Na^+^/K^+^ ratio manifested significant differences under the four salinity levels and among the BHS treatments (**Figure 1D**). Increasing salinity stress levels increased Na^+^/K^+^ ratio substantially. Compared with S1 level of salinity, Na^+^/K^+^ ratio showed a significant increase by 125.00, 177.78, and 205.56% under S2, S3, and S4, respectively. However, plants grown under the effect of 100, 150, and 200 mM NaCl and treated with T2 and T3 treatments of BHS depicted a significant decrease in Na^+^/K^+^ ratio. Under salt-free conditions (S1 level), T2 treatment decreased considerably Na^+^/K^+^ ratio, compared to the corresponding control.

### 3.3 Biochemical qualities

**Figure 2:**
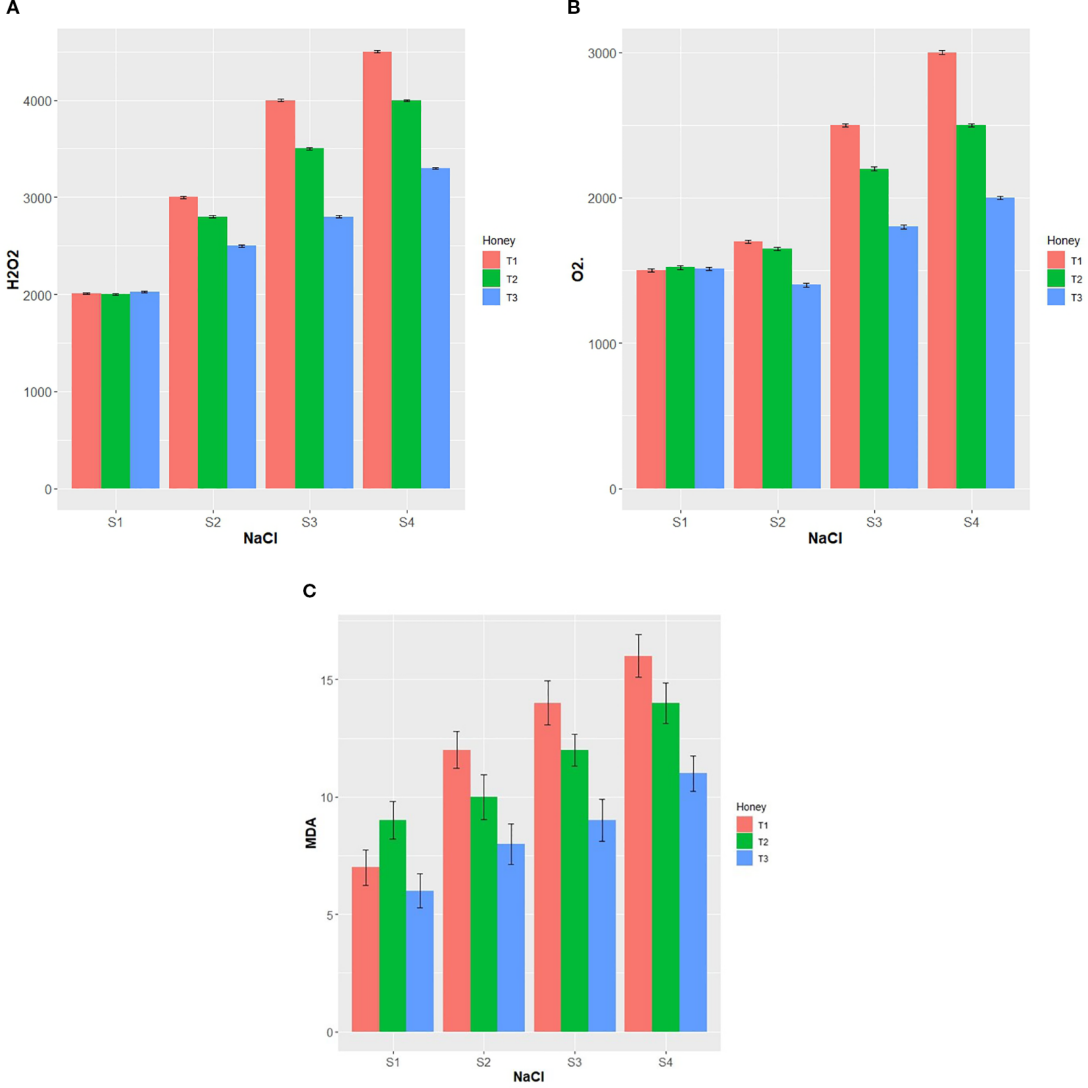
Interaction effect of foliar application with BHS treatments (T1: 0, T2: 1.0, and T3: 1.5%) and various levels of salinity (S1: 0, S2: 100, S3: 150, and S4: 200 mM NaCl) on reactive oxygen species and MDA of *Triticum aestivum* L., cv. Yecora Rojo plants. **(A)** H_2_O_2_, hydrogen peroxide (AU µg^-1^ protein), **(B)** O_2_•^−^, superoxide anion radicle (AU µg^-1^ protein), and **(C)** MDA, malondialdehyde content (nmol g^-1^ FW).

As exhibited in **Figures 2A, B**, the concentration values of reactive oxygen species (ROS), including O_2_•^−^ and H_2_O_2_ were elevated significantly with increasing concentrations of NaCl and reduced markedly by BHS supplementation. In plants subjected to S2, S3, and S4 of salinity, H_2_O_2_ was increased by 50.00, 100.00, and 125.00%, and O_2_•^−^ was elevated by 15.38, 53.85, and 100.00%, respectively, compared with the respective control. However, both T2 and T3 of BHS demonstrated a significant decrease in H_2_O_2_ and O_2_•^−^ under salinity levels from S2 to S4. Under unstressed conditions (S1 level), the S1 × T3 interaction recorded the lowest O_2_•^−^ concentration.

Results in **Figure 2C** manifest that MDA content of plants was increased significantly by rising salt concentrations. When salinity level increased from S1 (0 mM NaCl) to S4 (200 mM NaCl), the increase percentage of MDA was 128.57%. In contrast, rising concentrations of BHS decreased significantly MDA content. T2 and T3 treatments of BHS showed a significant decline in MDA concentration at S2 to S4 of salinity levels. Under S1, T3 decreased MDA content significantly.

**Figure 3:**
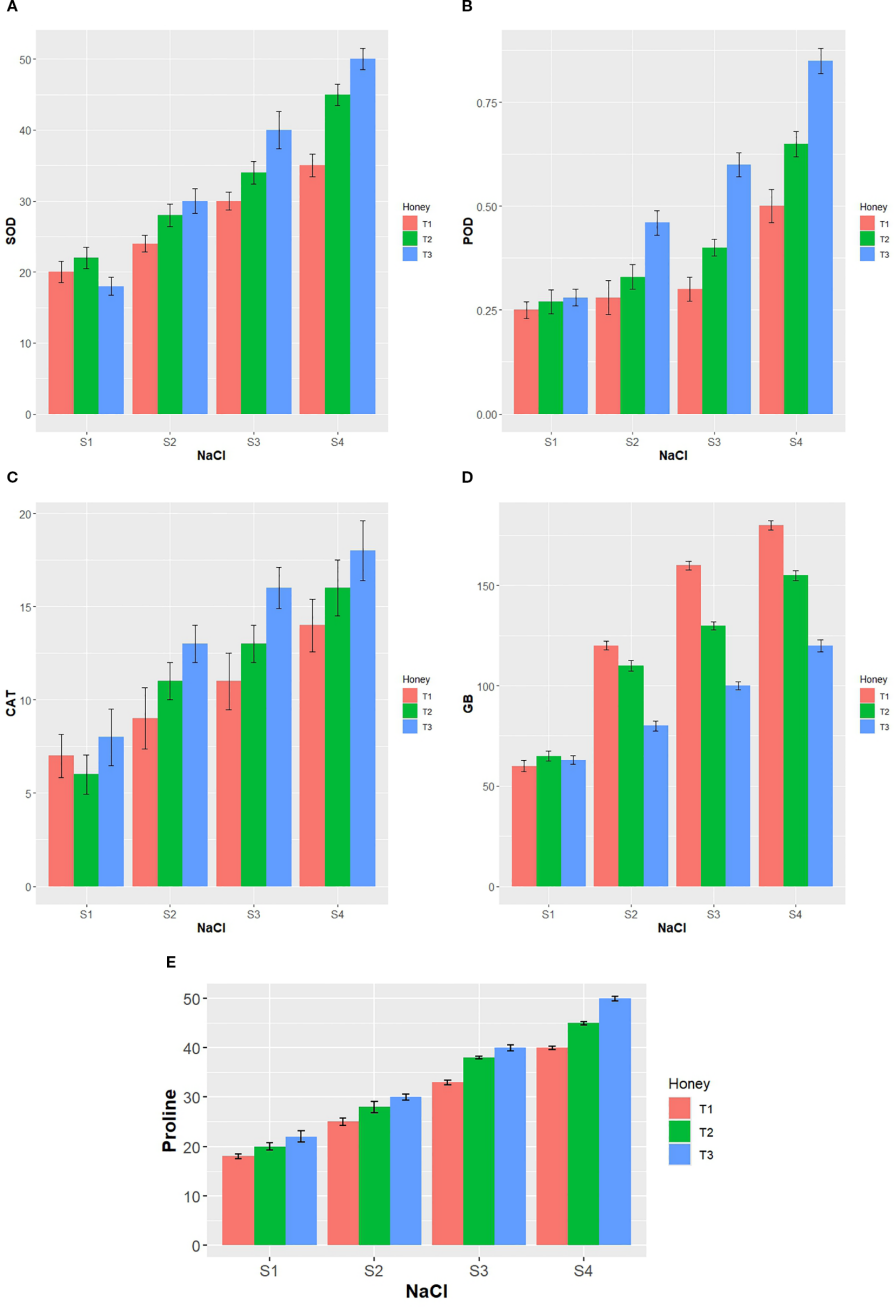
Interaction effect of foliar application with BHS treatments (T1: 0, T2: 1.0, and T3: 1.5%) and various levels of salinity (S1: 0, S2: 100, S3: 150, and S4: 200 mM NaCl) on antioxidant enzymes and osmolytes of *Triticum aestivum* L., cv. Yecora Rojo plants. **(A)** SOD, superoxide dismutase, **(B)** POD, peroxidase, **(C)** CAT, catalase (Enzyme units), **(D)** GB, glycine betaine (µg g^-1^ FW), and **(E)** proline (µg g^-1^ FW).

The catalytic activities of antioxidant enzymes SOD, POD, and CAT varied significantly in wheat plants due to combined influence of salinity and BHS treatments (**Figures 3A-C**). Plants exposed to salinity showed significant elevations in the activities of antioxidant enzymes. The S4 salinity level recorded SOD, POD, and CAT a marked increase of 66.67, 52.17, and 85.71%, respectively, compared to the corresponding control. However, applying BHS treatments (T2 and T3) enhanced substantially the activities of antioxidant enzymes. Both T2 and T3 of BHS treatments demonstrated significant increase in the catalytic activities of all antioxidant enzymes under S2 to S4 of salinity levels. The best treatment was T3, which recorded better enhancements in antioxidant enzymes activities under salinity conditions. Under S1 level of salinity, a significant increase in the activity of SOD was noted by the application of T2 treatment. Further, under the same salinity level (S1), POD activity was increased noticeably via T2 and T3 application, compared to the control.

Analyses of proline and GB showed significant differences due to the combined effect of salt stress and BHS application (**Figures 3D, E**). Increasing salinity stress levels increased proline and GB substantially. Under S4, the percentages of their increase were 122.22 and 193.10%, respectively. However, increasing BHS levels increased proline and minimized GB significantly. BHS treatments T2 and T3 depicted a significant increase in proline at all salinity levels, while they decreased GB under the effect of S2, S3, and S4 significantly. Under S1, both T2 and T3 increased GB significantly, compared with the control.

### 3.4 Gene expression analysis

**Figure 4:**
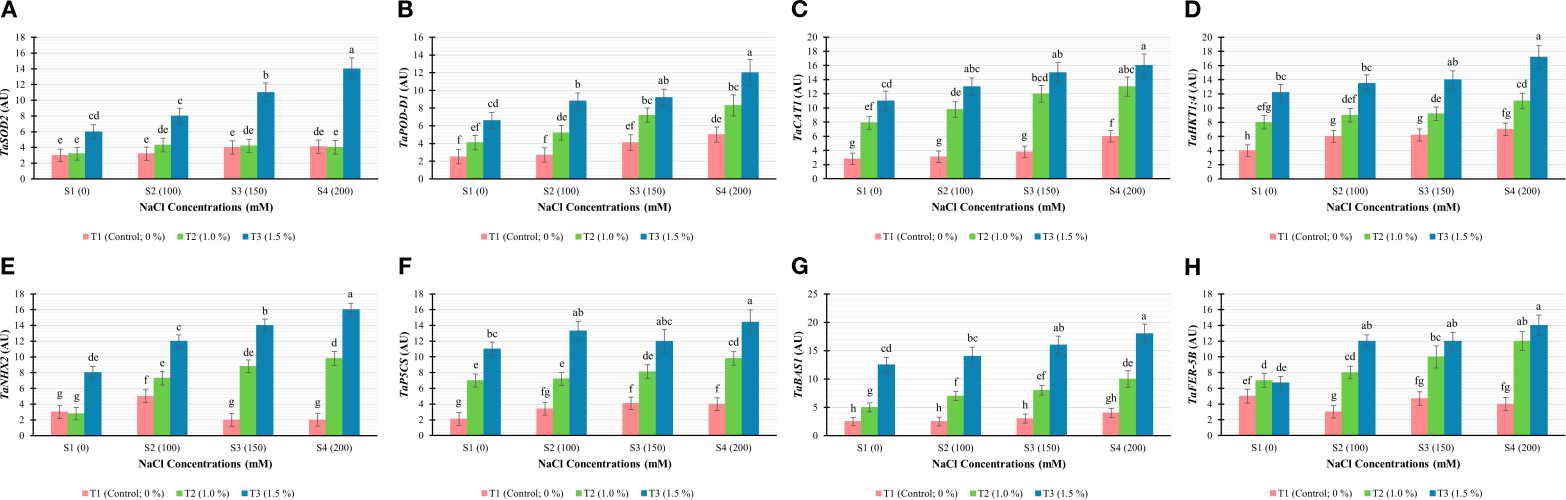
Relative expression of salinity-related genes in wheat (*Triticum aestivum* L., cv. Yecora Rojo) under the combined impact of salinity levels (S1: 0, S2: 100, S3: 150, and S4: 200 mM NaCl) and BHS treatments (T1: 0, T2: 1.0, and T3: 1.5%). **(A)***TaSOD2*, **(B)***TaPOD-D1*, **(C)***TaCAT1*, **(D)***TaHKT1;4*, **(E)***TaNHX2*, **(F)***TaP5CS*, **(G)***TaBAS1*, and **(H)***TaFER-5B*. Means ± SD labelled by different letters are significantly different at *P* ≤ 0.05.

The expression of salinity-related genes for instance *TaSOD2*, *TaPOD-D1*, *TaCAT1*, TaHKT*1;4*, *TaNHX2*, *TaP5CS*, *TaBAS1*, and *TaFER-5B* was affected by the varying concentrations of BHS under all salinity stress levels (**Figures 4A-H**). Relative expression of the gene *TaSOD2* depicted a significant (*P* ≤ 0.05) difference under salinity levels due to varying concentrations of BHS in comparison to control. At all concentrations of NaCl, the highest level of *TaSOD2* transcripts was discovered at BHS level T3, while the lowest transcripts level was recorded at T1. Correspondingly to *TaSOD2* gene, *TaPOD-D1* and *TaCAT1* showed significant (*P* ≤ 0.05) different expression under varying salinity levels due to changing concentrations of BHS as compared to control. At all concentrations of NaCl, the genes *TaPOD-D1* and *TaCAT1* manifested maximum expression at BHS treatment T3 followed by T2, and the minimum relative expression was recorded at T1. Generally, the expressions of *TaSOD2*, *TaPOD-D1* and *TaCAT1* were in parallel with the enzymatic activities of SOD, POD and CAT, respectively.

*TaHKT1;4* and *TaNHX2* genes showed significant (*P* ≤ 0.05) varying levels of transcript due to the interactions of salinity and BHS treatments. At all concentrations of salinity, the maximum increase in the expression of *TaHKT1;4* and *TaNHX2* was recorded at T3 of BHS treatments while the minimum level was reported at T1. Furthermore, the upregulation of *TaHKT1;4* and *TaNHX2* genes was in consistent with the K^+^ influx at corresponding concentrations of salinity and BHS as indicated in Na^+^/K^+^ ratio.

Regarding *TaP5CS* gene, its relative expression depicted significant (*P* ≤ 0.05) difference under all salinity concentrations due to varying BHS treatments. The maximum *TaP5CS* expression was recorded at T3 followed by T2 and the minimum expression was recorded at T1. For the analysis of *TaBAS1* and *TaFER-5B* genes, their expression differed significantly (*P* ≤ 0.05) across all salinity levels due to the varying BHS treatments. The maximum relative expression of *TaBAS1* gene was recorded at T3 level of BHS followed by T2 at all levels of salinity. In case of *TaFER-5B* gene, T3 treatment of BHS followed by T2 recorded the highest expression of the gene at salinity levels from S2 to S4. Under S1 level of salinity, the maximum expression of *TaFER-5B* gene was recorded at T2 treatment. Further, the expression of *TaBAS1* and *TaFER-5B* was in consistent with the scavenging activity against H_2_O_2_ and O_2_•^−^.

### 3.5 Correlation analysis

**Figure 5:**
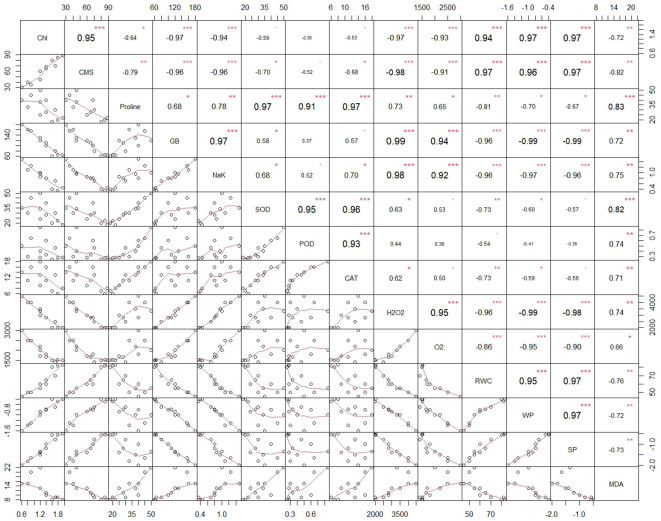
Overall correlation among different traits of *Triticum aestivum* L., cv. Yecora Rojo plants at four levels of salinity stress (0, 100, 150, and 200 mM NaCl) due to BHS different treatments (0, 1.0 and 1.5%). The Pearson coefficients are shown in the upper matrix, and results were significant at *P* < 0.1 (*), *P* < 0.05 (**), and *P* < 0.01 (***). Regression between two traits represented by the red solid lines in the lower matrix of the figure.

The overall correlation analysis of antioxidant enzymes, chlorophyll, CMS, MDA, ROS, proline, GB, Na^+^/K^+^, and plant water relations has clarified a significant and varying extent of paired association among them. Generally, both type and level of treatments altered the significance of paired association among traits. The chlorophyll manifested a strong positive paired association with CMS, RWC, Ψ_w_, and Ψ_s_ and negative paired association with proline, GB, Na^+^/K^+^, MDA, and ROS (H_2_O_2_ and O_2_•^−^) (**Figure 5**). Similarly to chlorophyll, the CMS varied in opposite direction with proline, GB, Na^+^/K^+^ ratio, MDA, ROS, and the antioxidant enzymes SOD and CAT. Moreover, RWC, Ψ_w_, and Ψ_s_ depicted negative association with osmolytes, antioxidant enzymes, MDA, and ROS. In general, there was a positive association among the activity of antioxidant enzymes (POD, SOD, and CAT), ROS, MDA, and osmolytes.

**Figure 6:**
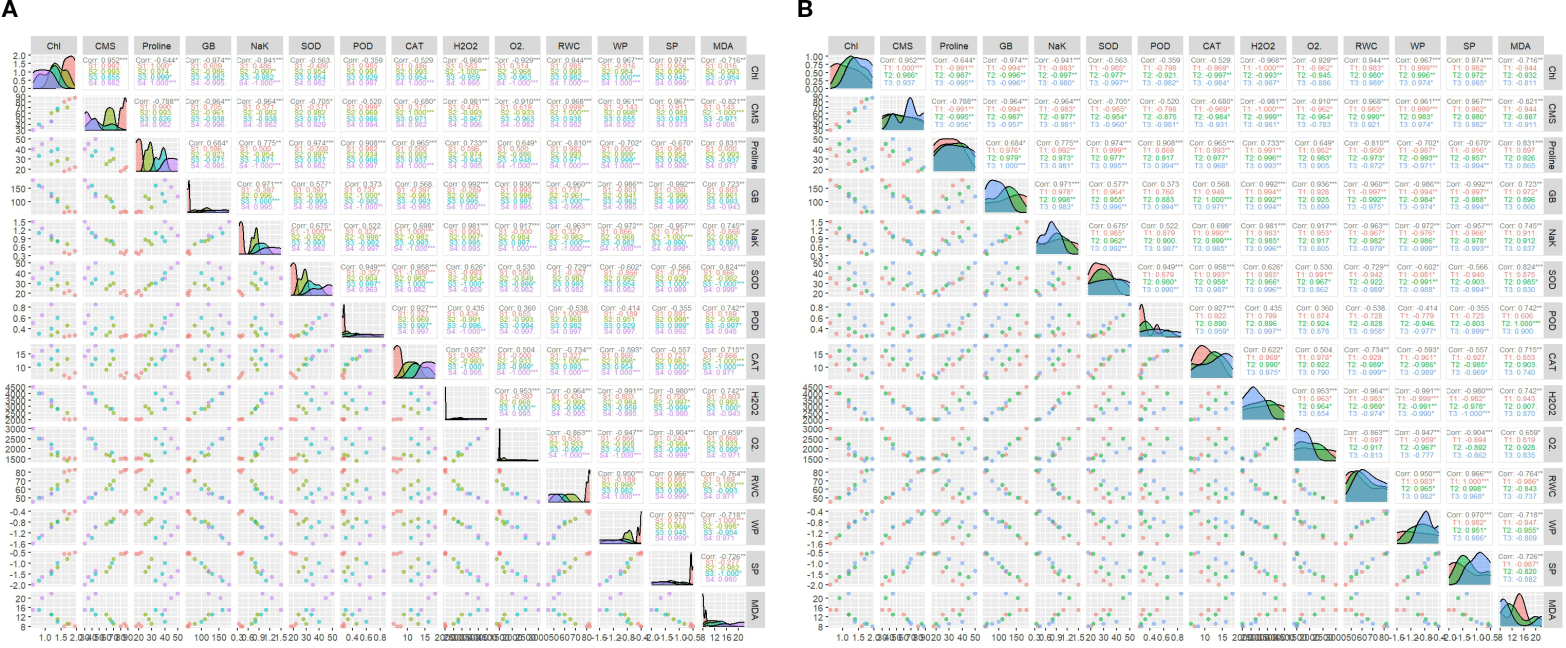
Pearson correlation among different traits of *Triticum aestivum* L., cv. Yecora Rojo **(A)** due to the effect of salinity treatments (S1: 0, S2: 100, S3: 150, and S4: 200 mM NaCl), **(B)** due to the effect of BHS treatments (T1: 0, T2: 1.0, and T3: 1.5%). (* Significant at *P* ≤ 0.1, ** significant at *P* ≤ 0.01, and *** significant at *P* ≤ 0.001).

Regarding the correlation analysis with respect to individual effect of salinity and BHS treatments, traits showed various ways of paired association. The correlogram analysis for the individual effect of salinity treatments pointed that all treatments affect the traits paired association in different ways which evidenced that the association of traits is highly vulnerable to the concentration of salinity (**Figure 6A**). Equivalently to salinity treatments effect, the impact of BHS treatments has proved that the paired association of traits is highly vulnerable to the concentrations of treatments (**Figure 6B**).

**Figure 7:**
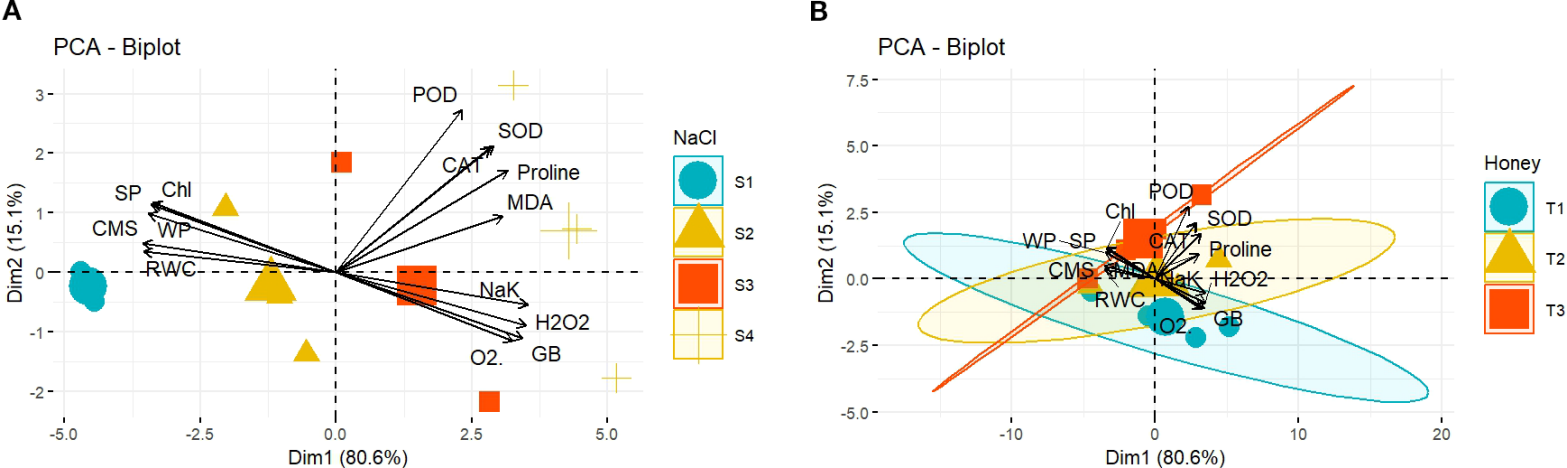
PCA scatter plot graph showing the clustering of biochemical and physiological traits of *Triticum aestivum* L., cv. Yecora Rojo plants. **(A)** as they were influenced by salinity concentrations (S1: 0, S2: 100, S3: 150, and S4: 200 mM NaCl), **(B)** in relation to BHS treatments (T1: 0, T2: 1.0, and T3: 1.5%).

The PCA biplot analysis for salinity treatments clarified that every salinity concentration (0, 100, 150, and 200 mM NaCl) affected differently the extent of association and expression of antioxidant enzymes, chlorophyll, CMS, MDA, ROS, proline, GB, Na^+^/K^+^, and plant water relations (**Figure 7A**). Equivalently, the biplot analysis for the efficacy of BHS treatments (0, 1.0, and 1.5%) also proved that the varying levels of BHS affected the response of the traits differently (**Figure 7B**). The BHS concentrations distributed varyingly in biplot quadrants which evidences that the variation in the degree of traits correlation is extremely associated with the levels of BHS. As **Figure 7** shows, the most affected parameters were POD, SOD, and CAT as they have the longest arrows and are aligned with the Dim1 axis, which illustrates most of the variance. Generally, among salinity and BHS treatments, S4 level of salinity (200 mM NaCl) and T3 concentration of BHS (1.5%) have the most impact on the parameters as they are away from the biplots origin and have large arrows pointing to them.

### 3.6 Heatmap analysis

**Figure 8:**
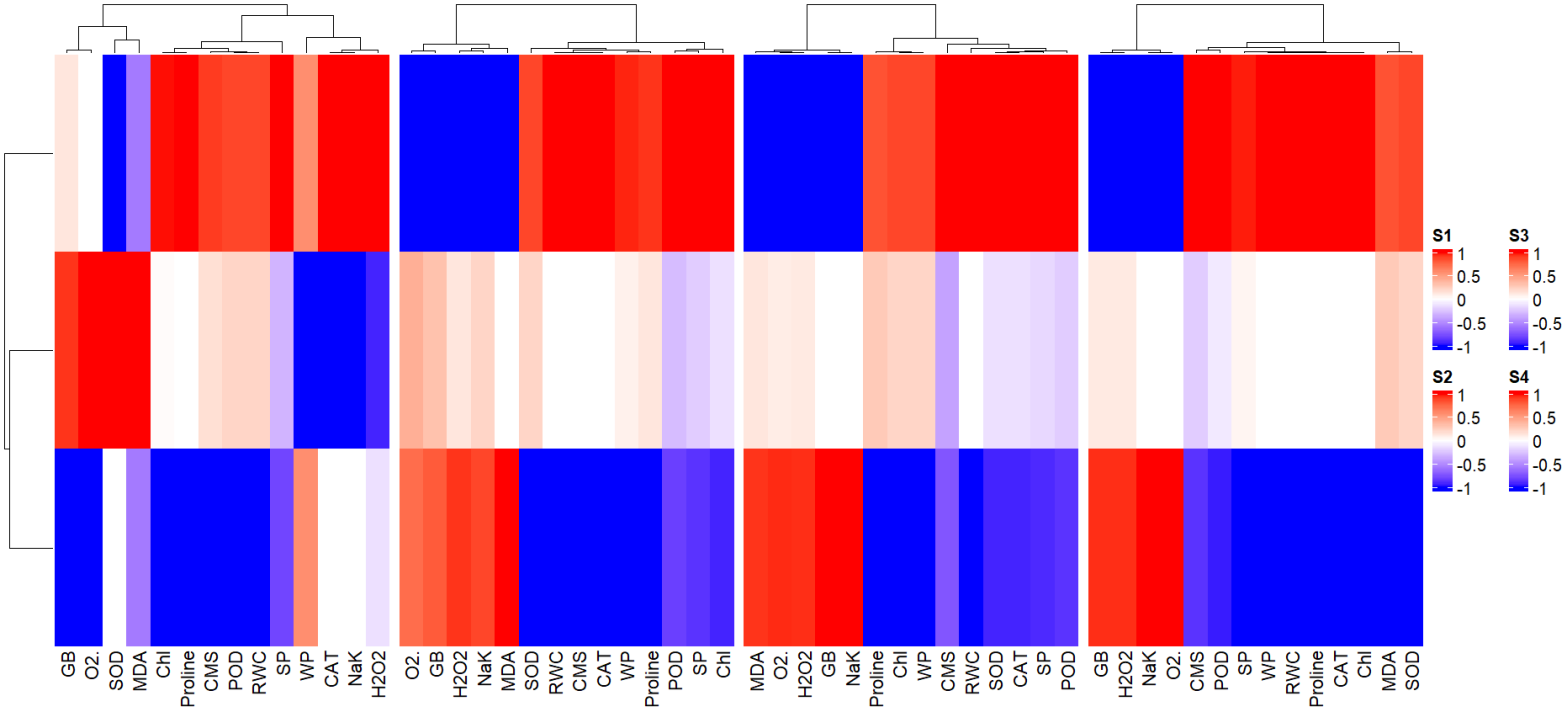
Cluster heatmap indicating the combined impact of salinity and BHS (salinity × BHS) treatments on traits response and correlation. The bottom row represents the T1 (0%) treatment across different salinity levels (S1: 0, S2: 100, S3: 150, and S4: 200 mM NaCl), T3 (1.5%) is shown in the top row while T2 (1.0%) lies in between.

The disparate band patterns and clusters allocation of heatmap pointed out that expression and association of antioxidant enzymes, chlorophyll, CMS, MDA, ROS, proline, GB, Na^+^/K^+^, and plant water relations are different for same levels of BHS (0, 1.0, and 1.5%) under different concentrations of salinity stress (0, 100, 150, and 200 mM NaCl) as displayed in **Figure 8**. This has evidenced that varying interactions of salinity and BHS (salinity × BHS) affect the correlation and expression of traits in various ways.

## 4 Discussion

Salinity is one of the most hazardous abiotic stresses limiting wheat global productivity by impairing water uptake, physiological processes, biochemical activities, and antioxidant balance. Plants under salt stress experience excessive Na^+^ accumulation, reduced K^+^/Na^+^ ratio, and oxidative damage. To mitigate these effects, biostimulants have emerged as eco-friendly alternatives that increase plant resilience by modulating physiological and molecular processes.

In the current study, increasing salinity level from S1 (0 mM NaCl) to S2 (100 mM NaCl), S3 (150 mM NaCl), and S4 (200 mM NaCl) increased Na^+^/K^+^ ratio, restricted plants growth (**Table 3**), reduced chlorophyll, and impeded leaf integrity (e.g., CMS and RWC) (**Figure 1**). These negative impacts were due to the induction of lipid peroxidation (MDA) as a consequence of ROS (H_2_O_2_ and O_2_•^−^) excessive generation (**Figure 2**). These adverse salinity-induced outcomes were linked with an increase in osmo-protectant compounds and upregulation of antioxidant enzymes activity (**Figure 3**). These findings were in agreement with numerous previous studies (Azzam et al., 2021; Atta et al., 2023). Salinity-exacerbated outcomes can be ascribed to the osmotic stress which causes loss of the cell turgor, and the oxidative stress associated with the overproduction of ROS (Upadhyaya et al., 2013).

The present study found that the growth indices of wheat were significantly improved via leafy application of BHS under both normal and saline conditions (**Table 3**). Enhancement of plants growth by BHS could be attributed to its high contents of essential nutrients, proline, and soluble sugars which are pivotal for the formation of cellular protoplasm (Lemoine et al., 2013). Additionally, phytohormones content of BHS encompassing cytokinins and auxins are accountable for rapid cell multiplication, enlargement, and division (Wang et al., 2017; Sosnowski et al., 2023). In plants treated with bio-stimulants, metabolic pathways stimulation along with phenylpropanoid synthesis may interpret the relieving of stress influences on plants (Ertani et al., 2013).

In the present work, reduction of chlorophyll concentration under salinity levels (S2, S3, and S4) (**Figure 1A**) is owing to the overmuch ROS production induced by salinity stress (**Figures 2A, B**) which stimulated chlorophyll degradation and chloroplast chlorosis. These findings mirror those reported by Muhammad et al. (2021) and Rady et al. (2021). Nevertheless, BHS has a protective role on the photosynthetic systems (Semida et al., 2019). In the current work, chlorophyll content was restored in salinity-stressed plants by BHS foliar spray (**Figure 1A**). This finding was positively correlated with CMS maintenance and RWC restoration by BHS application (**Figure 6B**). Possibly, wheat plants responded effectively to salt stress through several mechanisms of BHS, including mineral nutrient balance (in terms of more K^+^ influx) as well as upregulation of various antioxidant enzymes (**Figures 3A-C**) to minimize excess ROS (H_2_O_2_ and O_2_•^−^), thereby reducing peroxidation of lipids (MDA) as well as electrolyte leakage (EL). Nutrients content of BHS maintains the intracellular ion homeostasis necessary for chlorophyll biosynthesis, thus activating photosynthesis process and consequently more assimilates are produced. These substances along with osmoregulatory compounds of BHS improve the osmotic adjustment of the plant cells by increasing RWC and cell turgidity to maintain a healthy cellular medium for metabolic processes (Alghamdi et al., 2022).

In metabolizing tissues, RWC, Ψ_w_, and Ψ_s_ are physiological indicators of the water status (Yan et al., 2016), as for membrane integrity degree, it can be estimated as CMS and EL (Zhao et al., 2017). With respect to the water status of plants under S2, S3, and S4 levels of salinity, it was found that salinity reduced Ψ_s_, Ψ_w_, and RWC as illustrated in **Figures 1C, E, F**. This result may be interpreted by the fact that excessive salts in the soil lower the osmotic potential, which in turn declines the overall water potential and adversely affects water uptake by plants. Consequently, osmotic stress is induced in plants which causes stomatal closure and hinders the shoot growth (Chele et al., 2021). In response to these salinity-induced effects, plants may alter their metabolism by the hyperaccumulation of metabolites as one of several mechanisms support to withstand salinity. Hence, the osmotic potential of plants is decreased under salt stress, which subsequently reduces water potential and RWC. Since solute accumulation is proportional to the rate of decline in plant water status (Blum, 2017), stressed plants treated with BHS in the present study showed less negative Ψ_s_ values compared to un-stressed plants. This confirms that BHS played a role in alleviating the negative impacts of salinity on plant water status, thereby maintaining higher water content. Furthermore, past studies have reported that foliar application of BHS triggers root activity and increases root fresh and dry weights in stressed plants (Abou-Sreea et al., 2021; Tarfayah et al., 2023). Such modifications likely expand root absorption surface, therefore enhancing nutrient uptake under stress and supporting ionic balance to maintain cell turgor and ultimately favoring higher RWC (Alghamdi et al., 2022).

Ion toxicity, which results from the deposition of higher Na^+^ in plant cells, is one of the major detrimental impacts of salinity. Excess Na^+^ is highly damaging to plants, as it can induce cytosolic K^+^ efflux through ROS signaling, thereby, perturbing cellular homeostasis. This leads to interference with K^+^ and Ca^2+^ functions, nutrient deficiencies, induction of oxidative stress, and growth retardation (Assaha et al., 2017). The findings of the current study are consistent with these well-established effects. Besides, increasing root K^+^ uptake and increasing external K^+^ availability can overcome salinity-induced damage in plants (Rodrigues et al., 2013). In the current work, wheat plants treated with BHS under salt stress showed a marked decrease in the Na^+^/K^+^ ratio (**Figure 1D**). Since BHS is rich in essential minerals, including K^+^ (**Table 1**), thereby its foliar application may act as an exogenous K^+^ supplement under stress. In addition, by increasing root growth and absorption surfaces, BHS likely triggered the tendency of roots to take up K^+^. Furthermore, BHS antioxidant activity, through reducing ROS levels, may have stopped K^+^ efflux from root cells. Overall, these effects allowed BHS-treated plants to maintain higher cytosolic K^+^ as compared to stressed untreated plants.

Proline accumulation is generally considered as a characteristic plant response to multiple abiotic stresses, including salinity as explained by Koc et al. (2024). In this study, wheat plants exposed to salt stress (S2, S3, and S4) exhibited a dynamic increase in proline and glycine betaine (GB) contents (**Figures 3D, E**). Both osmo-protectants play vital protective roles against abiotic stress by maintaining cell turgor, regulating osmotic balance, and scavenging ROS. Proline specifically imparts osmotic homeostasis and protection against oxidative stress, while GB functions as an osmolyte, antioxidant activator, and Na^+^ toxicity mitigator (Zhu et al., 2022). Interestingly, in present study, BHS treatment enhanced proline but declined GB accumulation compared with stressed untreated plants (**Figure 3**). Elevated proline content indicates that BHS activated stress response pathways pointing to the versatile role of BHS in stress resilience. Benito et al. (2023) reported similar GB results, where potassium humate enhanced stress tolerance in *Arabidopsis thaliana*.

Salt stress promotes ROS generation, hence necessitating an upregulation of enzymatic antioxidants for plant survival as stated by Abogadallah (2010). In line with this, activities of SOD, POD, and CAT in stressed plants increased with rising salt concentrations (100, 150, and 200 mM; **Figures 3A-C**). Besides, under slat stress, reduced stomatal conductance (Gs) limits CO_2_ uptake, slowing Calvin cycle and causing NADP^+^ depletion in photosystem I that results in the overproduction of O_2_•^−^ and H_2_O_2_ due to photorespiration in C_3_ plants (Abogadallah, 2010; Hasanuzzaman et al., 2021). Excess of ROS causes oxidative damage to biomolecules, leading to lipid peroxidation, protein oxidation, and DNA degradation (Sharma et al., 2012). Consistence with these findings, the present study recorded increased levels of H_2_O_2_, O_2_•^−^, and MDA in salt-stressed plants (**Figures 2A-C**). Importantly, BHS treatment enhanced antioxidant enzyme (SOD, POD, and CAT) activities, reducing ROS-inducing damage. This modulation may be associated to the over-expression of *TaSOD2*, *TaPOD-D1*, and *TaCAT1* genes (**Figures 4A-C**), along with increased antioxidant activities.

Relative gene expression analysis revealed strong effects of combined salt and BHS treatments. Under salt stress, untreated plants showed the upregulation of *TaSOD2*, *TaPOD-D1*, and *TaCAT1*, confirming their role in oxidative stress tolerance. Besides, expression of *TaBAS1* was also enhanced, while *TaFER-5B* and *TaNHX2* illustrated varying responses based on NaCl level. *TaP5CS* and *TaHKT1;4* were consistently triggered by salinity, suggesting their roles in proline biosynthesis and K transport, respectively. Notably, BHS treatment significantly upregulated all stress associated genes under both control and stress conditions, with the highest effects at 1.5% BHS (T3 treatment; **Figure 4**). This indicates that BHS increases transcriptional regulation of salt stress-responsive genes. Besides, earlier studies have shown parallel outcomes, where upregulation of *TaSOD2* increased ROS-scavenging enzyme SOD activities (Wang et al., 2016), and upregulation of *TaPOD-D1* and *TaCAT1* improved POD and CAT activity (Geng et al., 2019; Sun et al., 2024). Likewise, *TaHKT1;4* and *TaNHX2* modulate Na^+^/K^+^ transport to maintain ionic homeostasis (Gholizadeh et al., 2024; Li et al., 2024). Genes *TaBAS1* and *TaFER-5B* contribute to ROS detoxification as reported by Xiao et al. (2023) and Zang et al. (2017), while *TaP5CS* is essential for proline biosynthesis via the glutamate pathway as reported by Aycan et al. (2022). The parallelism between gene upregulation and improved physiological and biochemical traits (**Figures 1**–**3**) confirms the efficacy of BHS in enhancing salt stress tolerance.

Plants’ response to environmental conditions and/or external stimulants is a complex process including several mechanisms. The molecular mechanism of this process is a multi-level process involving sensing, signal transduction, transcription, processing and protein translation and modification. It is a complex response mechanism with multiple genes, signaling pathways, and metabolic processes (Zhang et al., 2023). Previous studies have reported that sugars, minerals, and antioxidants, which are the key components of BHS, act as crucial signals, triggering transcriptional regulation of plants by affecting secondary messengers such as Ca^2+^, altering hormone pathways, and activating specific transcriptional factors (TFs), thereby controlling gene expression for growth and stress defense (Kehr, 2013; Hürkan and Hürkan, 2025).

Correlation analysis further confirmed these findings. Positive paired association among MDA, H_2_O_2_, O_2_•^−^, GB, proline, and antioxidant enzyme (SOD, POD, CAT) activities (**Figures 5**, **6**) represents the complex interplay of oxidative stress and defense mechanisms under salt stress (Hasanuzzaman et al., 2021). Furthermore, strong positive correlations among RWC, Ψ_w_, and Ψ_s_ suggested improved plant water relations under BHS treatment, while negative correlations between Na^+^/K^+^ ratio and photosynthetic or structural traits (chlorophyll, CMS) indicated the ion toxicity (Assaha et al., 2017; Muhammad et al., 2021).

Multivariate analyses further explained the treatment effects. PCA biplots (**Figures 7A, B**) depicted clear clustering of salt levels (S1–S4), indicating differential plant responses to each salinity treatment or level. Among BHS treatments, T3 illustrated the most distinct clustering, highlighting its strong influence on plant tolerance and resilience. Similarly, heatmap analysis showed that severe salinity (S3, S4) caused dramatic oxidative stress and physiological damage in untreated plants (**Figure 8**). By contrast, plants treated with BHS, especially at T3, showed declined ROS, higher antioxidant activities, and improved proline accumulation. Together with enhanced gene expression, these results prove that BHS application at 1.5% indicates an effective bio-stimulant application strategy to mitigate salt stress in wheat. A comparative summary of previous bio-stimulants and the present study’s BHS is presented in **Table 4**. Unlike conventional bio-stimulants that chiefly target single stress pathways, BHS represents a multi-functional role by improving ionic homeostasis, osmotic adjustment, and antioxidant defense in wheat under salinity stress.

**Table 4:** A comparative summary of previous biostimulants and the current study’s bee-honey solution (BHS).

Biostimulant type	Crop	Mechanisms	Key benefits under stress	Citation
Salicylic acid (SA)	Wheat (under salt stress)	Activates antioxidant response	Improved germination and growth parameters (at mild and moderate stress)	Fardus et al. (2018)
Seaweed extract (from *Ulva rigida*)	Wheat (under salt stress)	Improves antioxidant enzyme activities	Increased growth and photosynthetic pigments	Latique et al. (2021)
Salicylic acid, gibberellic acid, and cytokinins (combination)	Wheat (under salinity and drought stress)	Balance development under stress	Increased grain yield traits	Tajdari et al. (2024)
ZnO nanoparticles foliar application	Pea (under salinity stress)	Reduces oxidative stress and improved growth	Reduced H_2_O_2_ and MDA, and improved root growth	Mustafa et al. (2024)
Reduced graphene oxide nanoparticles (rGO) NPs	Wheat (under lead stress)	Suppress ROS production	Increased chlorophyll content and antioxidant enzyme activities	Zhan et al. (2024)
Iron oxide nanoparticles (Fe_2_O_3_-NPs)	Wheat (under chromium stress)	Improve antioxidant enzyme activities	Improved morphological and physiological responses	Zafar et al. (2024)
**Current Study: Bee Honey Solution (BHS)**	Wheat cv. Yecora Rojo (under salinity)	Improves physiological, morphological, biochemical, and genetic mechanisms	Improved salt tolerance through combined physiological, biochemical and genetic mechanisms	Present study

Overall, findings of the present study provide reasonably consistent evidence of BHS efficacy in alleviating the noxious impacts of salt stress in wheat plants. These outcomes outline the critical role of BHS foliar application in improving plants resilience to salt stress which can be applied to plants stressed with salinity under field conditions.

## 5 Conclusion

The present study has explored the exogenous application influence of BHS on *T. aestivum* cv. Yecora Rojo plants grown under normal conditions or stressed with salinity. The morphological, physio-biochemical and molecular responses of plants revealed that salt stress was impediment to the plant growth through increasing Na^+^/K^+^ ratio, oxidants (H_2_O_2_ and O_2_•^−^), and oxidant damage affecting negatively tissue integrity, chlorophyll content, and RWC. However, exogenously applied BHS (1.5% as the best treatment) effectively alleviated these salt-induced adverse effects via increasing proline content, enzymatic antioxidant activities, and gene expression.

The present study has contributed to a better understanding of cv. Yecora Rojo responses to salt stress by analyzing the expression of various salt stress-affined genes. Likewise, the current findings on BHS effects under salt stress highlighted its beneficial role in mitigating salt stress impacts and enhancing salt stress tolerance. Overall, the results confirmed that BHS serves as an effective natural multi-stimulator for plants under both normal and stress circumstances.

## Data Availability

The datasets presented in this study can be found in online repositories. The names of the repository/repositories and accession number(s) can be found in the article/Supplementary Material.
